# First-in-human prospective trial of sonobiopsy in high-grade glioma patients using neuronavigation-guided focused ultrasound

**DOI:** 10.1038/s41698-023-00448-y

**Published:** 2023-09-16

**Authors:** Jinyun Yuan, Lu Xu, Chih-Yen Chien, Yaoheng Yang, Yimei Yue, Siaka Fadera, Andrew H. Stark, Katherine E. Schwetye, Arash Nazeri, Rupen Desai, Umeshkumar Athiraman, Aadel A. Chaudhuri, Hong Chen, Eric C. Leuthardt

**Affiliations:** 1 Department of Biomedical Engineering, Washington University in St. Louis, Saint Louis, MO 63130 USA; 2 Department of Pathology and Immunology, Washington University School of Medicine, Saint Louis, MO 63110 USA; 3 Mallinckrodt Institute of Radiology, Washington University School of Medicine, Saint Louis, MO 63110 USA; 4 Department of Neurosurgery, Washington University School of Medicine, St. Louis, MO 63110 USA; 5 Department of Anesthesia, Washington University School of Medicine, St. Louis, MO 63110 USA; 6 Department of Radiation Oncology, Washington University School of Medicine, Saint Louis, MO 63108 USA; 7 Department of Genetics, Washington University School of Medicine, St. Louis, MO 63110 USA; 8 Department of Computer Science and Engineering, Washington University in St. Louis, Saint Louis, MO 63130 USA; 9 Siteman Cancer Center, Washington University School of Medicine, St. Louis, MO 63110 USA; 10 Division of Neurotechnology, Department of Neurosurgery, Washington University School of Medicine, Saint Louis, MO 63110 USA; 11 Department of Neuroscience, Washington University School of Medicine, Saint Louis, MO 63110 USA; 12 Center for Innovation in Neuroscience and Technology, Washington University School of Medicine, Saint Louis, MO 63110 USA; 13 Department of Mechanical Engineering and Materials Science, Washington University in St. Louis, Saint Louis, MO 63130 USA

**Keywords:** Tumour biomarkers, Biophysics

## Abstract

Sonobiopsy is an emerging technology that combines focused ultrasound (FUS) with microbubbles to enrich circulating brain disease-specific biomarkers for noninvasive molecular diagnosis of brain diseases. Here, we report the first-in-human prospective trial of sonobiopsy in high-grade glioma patients to evaluate its feasibility and safety in enriching plasma circulating tumor biomarkers. A nimble FUS device integrated with a clinical neuronavigation system was used to perform sonobiopsy following an established clinical workflow for neuronavigation. Analysis of blood samples collected before and after FUS sonication showed that sonobiopsy enriched plasma circulating tumor DNA (ctDNA), including a maximum increase of 1.6-fold for the mononucleosome cell-free DNA (cfDNA) fragments (120–280 bp), 1.9-fold for the patient-specific tumor variant ctDNA level, and 5.6-fold for the TERT mutation ctDNA level. Histological analysis of surgically resected tumors confirmed the safety of the procedure. Transcriptome analysis of sonicated and nonsonicated tumor tissues found that FUS sonication modulated cell physical structure-related genes. Only 2 out of 17,982 total detected genes related to the immune pathways were upregulated. These feasibility and safety data support the continued investigation of sonobiopsy for noninvasive molecular diagnosis of brain diseases.

## Introduction

Brain tumor diagnosis relies on neuroimaging by magnetic resonance imaging (MRI) and computed tomography (CT), followed by surgical resection or tissue biopsy for histological confirmation and genetic characterization. Alternative approaches to obtain information on a brain lesion without surgery include lumbar puncture and blood draw^1^. Lumbar puncture for cerebral spinal fluid-based liquid biopsy is uncomfortable and carries procedural risk, limiting its use for repeated testing. In contrast, blood-based liquid biopsy is a noninvasive, rapid, and inexpensive method to obtain highly relevant information about the tumor^2^. This approach detects circulating tumor-derived biomarkers, such as DNA, RNA, proteins, and extracellular vesicles shed by tumor cells. Circulating tumor DNA (ctDNA), which carries information about the dynamics of cancer-specific genetic alternations, is currently the most well-studied and validated biomarker for liquid biopsy^3^. Although blood-based liquid biopsy-guided personalized therapy has already entered clinical practice to guide the precision treatment of several cancers^4,5^, extending it to brain cancer remains challenging^6^. Brain tumor-derived circulating tumor biomarkers are generally detected only at low abundance and in a limited number of patients, which makes analysis difficult in routine clinical practice^7–9^. This low abundance is primarily due to the blood-brain barrier (BBB). This physical barrier prevents the transfer of brain tumor biomarkers into the peripheral circulation, resulting in low detection sensitivity^6,10^. Even when the BBB is disrupted in brain tumors, the release of tumor-specific biomarkers into the peripheral circulation remains limited^1^. Existing studies all focus on developiong advanced biomarker detection techniques, such as droplet digital PCR (ddPCR)^11^ and optimized next-generation sequencing (NGS)^12^. There is a critical need for techniques that overcome the BBB responsible for the sparsity of tumor-specific biomarkers.

Transcranial low-intensity focused ultrasound (FUS) in combination with intravenously injected microbubbles is a promising technique for noninvasive, spatially targeted, and reversible disruption of the BBB^13^. FUS can penetrate the skull noninvasively and focus on virtually any brain region with millimeter-scale accuracy. Microbubbles, traditionally used as blood-pool contrast agents for ultrasound imaging, amplify and localize FUS-mediated mechanical effects on the vasculature via FUS-induced cavitation (i.e., microbubble expansion, contraction, and collapse). Microbubble cavitation generates mechanical forces on the vasculature^14^, reversibly increasing the BBB permeability in the FUS-targeted brain region. Typically, the permeabilized BBB usually reseals after a few hours^15^ or a few days^16^. Recent clinical studies have demonstrated the feasibility and safety of FUS-mediated BBB opening for brain drug delivery in patients with brain tumor^17–21^, Alzheimer’s disease^22^, amyotrophic lateral sclerosis^23^, and Parkinson’s disease^24^.

We hypothesized that FUS-induced BBB opening enables “two-way trafficking” between the brain and bloodstream^25^. With FUS-mediated BBB opening, agents in the bloodstream can enter the brain for the treatment of brain diseases, while brain tumor-derived biomarkers can be released into the bloodstream for diagnostic access. We term this FUS-induced release of biomarkers into the bloodstream for blood-based liquid biopsy as sonobiopsy. Sonobiopsy opens the BBB at the spatially targeted brain location, releases tumor-derived biomarkers from precisely defined tumor locations into the blood circulation, and enables timely detection of biomarkers in the blood to minimize clearance. Our previous study provided compelling preclinical evidence that sonobiopsy enriched circulating RNA, DNA, and proteins in small and large animal models^25–28^. Recently, we found that sonobiopsy improved the detection sensitivity of glioblastoma (GBM) tumor-specific EGFRvIII mutation from 7.14 to 64.71% and TERT C228T from 14.29 to 45.83% in a mouse GBM model. It also improved the diagnostic sensitivity of EGFRvIII from 28.57 to 100% and TERT C228T from 42.86 to 71.43% in a porcine GBM model^29^. By retrospectively analyzing blood samples collected from FUS-mediated drug delivery clinical trials, Meng et al. provided preliminary clinical evidence that FUS-induced BBB opening increased the concentrations of circulating biomarkers, including cell-free DNA, neuron-derived extracellular vesicles, and brain-specific protein^30^.

The most widely used FUS device in current brain drug delivery clinical trials is the MRI-guided FUS system, ExAblate Neuro, from InSightec Inc. This system utilizes a hemispherical-shaped FUS transducer with 1,024 elements and an aperture of 30 cm^31^. It was initially designed for thermal ablation and has been approved by the United States Food and Drug Administration to treat essential tremors and tremor-dominant Parkinson’s disease. While this device can be adapted for sonobiopsy, it is expensive and requires MR-compatible hardware, MR scanner time, and extensive training to operate the device. Although neuronavigation-guided FUS devices were developed for drug delivery, they need a robotic arm for positioning heavy FUS transducers with large apertures and customized optical trackers to guide the positioning of the FUS transducer. FUS devices used for drug delivery require high spatial precision and a large treatment volume to deliver drugs to cover the whole diseased brain region efficiently. However, FUS devices for sonobiopsy do not need to deliver therapeutic drugs or cover the entire tumor. Affordable and easy-to-use FUS devices are needed for sonobiopsy to target specific regions inside the tumor for spatially targeted biomarker release.

In this study, we introduce a compact FUS device that was agile and seamlessly incorporated into established clinical neuronavigation systems. This device enables the sonobiopsy procedure to be performed using a clinical workflow similar to neuronavigation-guided tissue biopsy commonly used in clinical practice. A pilot prospective sonobiopsy clinical study was conducted on patients with high-grade glioma utilizing this device to evaluate the feasibility and safety of sonobiopsy. The outcomes of our study reveal that sonobiopsy successfully enriched patient-specific ctDNA in plasma samples without causing any observable tissue damage.

## Results

### Study design

**Fig. 1:**
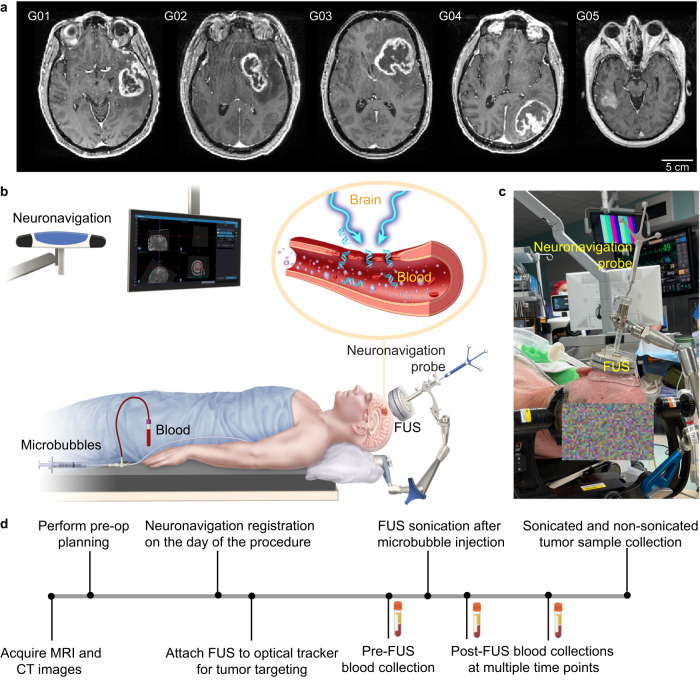
Sonobiopsy procedure. **a** Contrast-enhanced T1-weighted MRI images before sonobiopsy of five patients (G01, G02, G03, G04, and G05) enrolled in this study. **b** Illustration of the neuronavigation-guided sonobiopsy setup. **c** Picture of the FUS transducer coupled to the neuronavigation probe used during the sonobiopsy procedure. **d** Sonobiopsy clinical workflow.

This prospective single-arm trial aimed to assess the feasibility and safety of sonobiopsy in patients with high-grade glioma. The Washington University in St. Louis Institute Review Board approved the trial and registered with clinicaltrial.gov (Identifier: NCT05281731). Written informed consent was obtained from all participants before study enrollment. Patients with a lesion in the brain with imaging characteristics consistent with a high-grade glioma were screened for the clinical trial. Five patients (four men and one woman; average age 60 years; range 34–74 years) met the inclusion/exclusion criteria and were enrolled in the trial (Supplementary Table 1, Fig. 1a). Among them, four were GBM patients, and the other was a diffuse high-grade glioma patient. Details of the inclusion and exclusion criteria are provided in Supplementary Table 2. The primary outcome of this study was to evaluate the feasibility of sonobiopsy in enriching plasma ctDNA in post-sonication blood samples compared with pre-sonication blood samples. The secondary study outcome was to verify no evidence of brain tissue damage associated with the procedure.

### Sonobiopsy procedure was successful

**Fig. 2:**
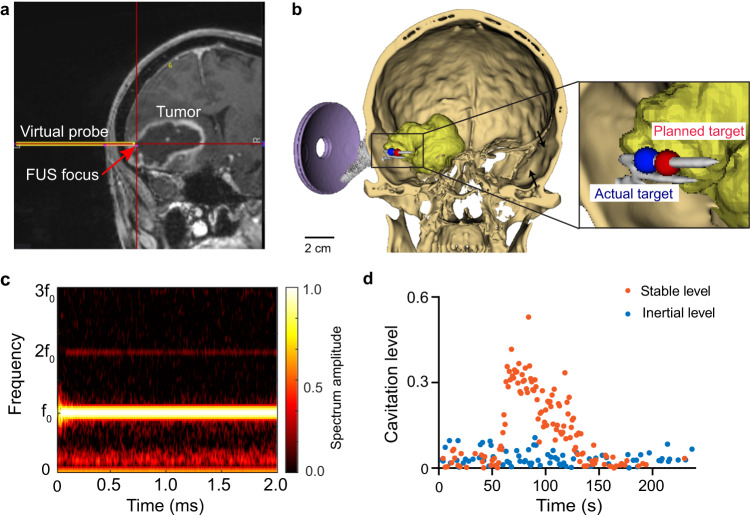
Tumor targeting and treatment monitoring. **a** The screenshot from the Stealth neuronavigation system shows the precise alignment of the FUS focus (arrow) with the planned target indicated by the crossing point of the red lines. **b** 3D reconstruction to show the spatial location of the planned target inside the tumor and the simulated FUS focus location based on the trajectory obtained from the neuronavigation system. **c** Representative time-frequency analysis of the acquired cavitation signals during FUS sonication to show the spectrum of the signals. **d** Representative stable cavitation and inertial cavitation levels measured based on the frequency spectrum of the acquired cavitation signals.

Sonobiopsy was performed after the patients were prepared for the surgery in the operating room and before the planned surgical removal of the brain tumor. Patients were under general anesthesia, and vital signs were continuously monitored by an anesthesiologist. Sonobiopsy was performed using a neuronavigation-guided FUS transducer (Fig. 1b, c). The procedure timeline is illustrated in Fig. 1d. MRI and CT images acquired before the procedure were loaded in the neuronavigation system (Stealth S8, Medtronic) and used for spatial registration of the patient’s head position. The patient’s hair above the tumor region was shaved. Degassed ultrasound gel was applied to the cleaned scalp for acoustic coupling. The FUS transducer with a water bladder attached was coupled to a standard neuronavigation probe with a customized adapter (Fig. 1c). The focus position of the FUS transducer was calibrated beforehand to be 80 mm from the tip of the stereotactic probe. An 80 mm offset was added in the neuronavigation software so that the tip of the “virtual probe” indicated the location of the FUS focus (Fig. 2a). The FUS transducer was mechanically positioned to align its focus at the planned tumor location. The acoustic pressure field was simulated based on the final trajectory of the probe, and the skull attenuation was estimated based on the simulation (Fig. 2b). The offset between the planned target and the simulated target location was found to be 1.89 ± 0.81 mm in the lateral direction and 4.21 ± 1.53 mm in the axial direction. The acoustic output pressure of the FUS transducer was adjusted to control the mechanical index (in situ acoustic pressure/square root of frequency) to be within 0.4–0.7 (Supplementary Table 3). Microbubbles (Definity, 10 µL/kg) were intravenously injected by an anesthesiologist, followed by FUS sonication for 3 mins. The FUS transducer had an acoustic sensor inserted in its center. Real-time cavitation monitoring provided an effective tool for monitoring the FUS sonication procedure (Fig. 2c, d) after injecting microbubbles. The stable and inertial cavitation levels were quantified based on the frequency spectrum of the acquired signals to quantify the bubble activity under stable oscillation (stable cavitation generates harmonic signals) and violent collapse (inertial cavitation generates broadband signals). The cavitation doses calculated by integrating the cavitation level over time for all five patients are summarized in Supplementary Table 3.

Blood samples were collected immediately before (5 min pre-FUS) and at different time points post-sonication (5, 10, and 30 min post-FUS). After the last blood collection, surgery was performed, and tumor tissue samples were collected from the FUS sonicated and nonsonicated tumor regions under the neuronavigation guidance. The total procedure time from when the patient was prepared ready to the end of FUS sonication was 22.2 ± 5.8 min.

### Sonobiopsy enriched plasma ctDNAs

**Fig. 3:**
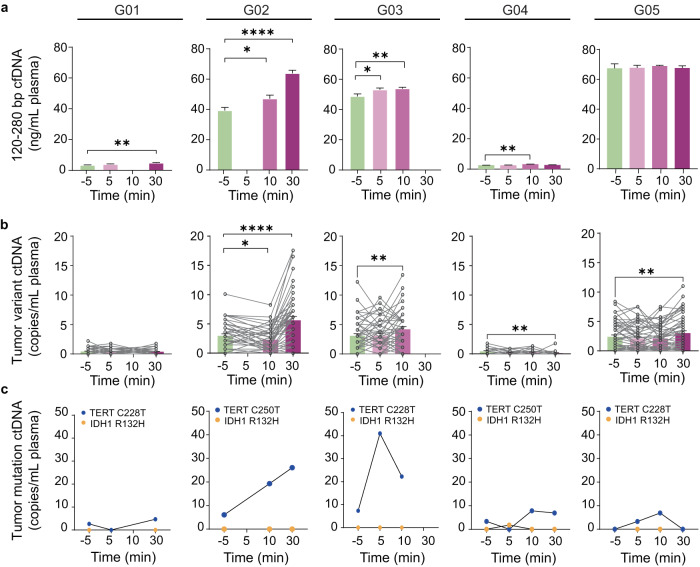
Sonobiopsy enriched plasma ctDNAs. **a** Plasma single nucleosome length cfDNA (120–280 bp fragments) concentrations in blood samples collected pre- and post-FUS sonication. The blood sample collection time points varied among patients. Bar graph represents mean ± standard error of the mean (SEM). *p < 0.05, **p < 0.01, ***p < 0.001, and ****p < 0.0001. Ordinary one-way ANOVA, uncorrected Fishers’ LSD comparing post-FUS time points with pre-FUS. **b** Plasma patient-specific tumor variant ctDNA concentrations measured using a personalized tumor-informed ctDNA assay. Bar graph represents mean ± standard error of the mean (SEM). *p < 0.05, **p < 0.01, ***p < 0.001, ****p < 0.0001. Repeated measures (RM) one-way ANOVA, uncorrected Fishers’ LSD comparing post-FUS time points with pre-FUS. **c** Plasma TERT and IDH1 mutation level detected by ddPCR. Data are presented for time points when the blood samples were collected.

We first analyzed the total cell-free DNA (cfDNA) levels in the plasma collected pre- and post-FUS at different time points (Supplementary Figure 1). Variations in the baseline concentrations of mononucleosome cfDNA fragments (120–280 bp) were observed among patients. Sonobiopsy increased the concentration of mononucleosome cfDNA fragments (120–280 bp) in post-FUS plasma samples from four patients (G01, G02, G03, and G04) compared with that in pre-FUS plasma samples (Fig. 3a). The highest plasma level of cfDNA for G01 and G02 was obtained at 30 min post-FUS with a 1.4-fold increase for G01 (*p* = 0.0027) and 1.6-fold increase for G02 (*p* = 7.51E−05) compared with that at pre-FUS. G03 and G04 reached the highest plasma level of cfDNA at 10 min post-FUS compared with that at pre-FUS with a 1.1-fold increase for G03 (*p* = 0.0115) and 1.3-fold increase for G04 (*p* = 0.0041).

The fraction of cfDNA derived from tumor cells is known as ctDNA. We utilized a personalized tumor-informed ctDNA assay (Invitae Personalized Cancer Monitoring assay, Invitae, San Francisco, CA) to assess the potential of sonobiopsy to improve the detection of patient-specific tumor variants in the plasma. This assay involved performing whole exome sequencing on the tumors and normal tissues. Sequencing results allowed the identification and selection of up to 50 tumor variants present in the tumors but not the matched normal tissues. The selected tumor variants were used in the design of a patient-specific panel, which was used to detect ctDNA in patient plasma samples. We compared the absolute level of patient-specific tumor variant ctDNA in the plasma samples (tumor variant ctDNA copies/ml plasma) collected before and after FUS. Our results showed that sonobiopsy enhanced the detection of patient-specific tumor variant ctDNA in the plasma of G02, G03, and G05 (Fig. 3b). Specifically, G02 reached the highest amount of tumor variant ctDNA at 30 min post-FUS compared with pre-FUS (5.58 ± 0.66 copies/mL at 30 min post-FUS vs. 2.93 ± 0.34 copies/ml at pre-FUS, 1.9-fold increase, *p* = 6.96E−07). G03 reached the highest level of plasma tumor variant ctDNA at 10 min post-FUS compared with pre-FUS (4.18 ± 0.45 copies/ml at 10 min post-FUS vs. 3.08 ± 0.38 copies/ml at pre-FUS, 1.6-fold increase, *p* = 0.0026). G05 reached the highest concentration of plasma tumor variant ctDNA at 30 min post-FUS compared with pre-FUS (3.02 ± 0.41 copies/ml at 30 min post-FUS vs. 2.39 ± 0.33 copies/ml at pre-FUS, 1.3-fold increase, *p* = 0.0047). No significant increase in patient-specific tumor variant ctDNA concentration in the plasma was observed for G01, whereas G04 showed a notable decrease after FUS treatment. However, the tumor variant levels were low for G01 and G04 throughout all time points.

A combination of TERT promoter mutation and IDH wildtype is the most common genotype observed in GBM. TERT promoter mutations are present in more than half of GBM patients and are associated with poor treatment outcomes^32,33^. IDH wildtype has become a diagnostic criterion for GBM since 2021 by WHO^34^. All five patients were IDH1 wildtype, and four patients (except for G05) were positive for TERT mutations based on analysis of resected tumor tissue (Supplementary Figure 2). To investigate the potential of sonobiopsy in detecting these two known mutations, we analyzed the amount of TERT mutation (C228T and C250T) and IDH1 mutation (R132H) in the plasmas with droplet digital PCR (ddPCR). The plasma levels of TERT mutations in post-FUS plasma samples were higher than that those in pre-FUS plasma samples for G02 and G03, with respective 3.2-fold and 4.3-fold increases at 10 min and 30 min post-FUS for G02, and 5.6-fold and 3.0-fold increases at 5 min and 10 min post-FUS for G03 (Fig. 3c). The detection of plasma TERT mulation ctDNA in G01 and G04 was limited (<10 copies/ml plasma). The TERT mutation level was low for G05 who was negative for TERT mutations. The IDH1 R132H ctDNA remained undetectable (<2 copies/mL plasma) in the plasma regardless of FUS treatment (Fig. 3c).

### Sonobiopsy did not induce detectable tissue damage

**Fig. 4:**
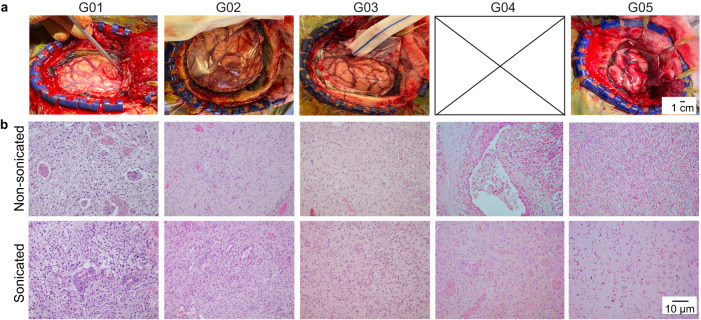
Safety of sonobiopsy by tissue analysis. **a** The gross pathology examination of brain surface after craniotomy for the four patients (G01, G02, G03, and G05) did not observe tissue damage induced by the FUS procedure. Piciture of G04 was not captured. **b** Hematoxylin and eosin (H&E) staining of the sonication and nonsonicated brain tumor tissue did not observe clear evidence of tissue damage.

During the FUS sonication procedure, we did not observe any significant fluctuations in vital signs, such as heart rate and respiration, nor did we note any adverse events. Following FUS sonication, we did not observe any signs of hemorrhage on the brain’s surface caused by the FUS procedure (Fig. 4a). Additionally, the sonicated and nonsonicated tumor regions were dissected and collected at 1.7 ± 0.4 h after FUS sonication. Both sonicated and nonsonicated tumor tissues were stained by hematoxylin and eosin and evaluated by a neuropathologist who was blinded to the study (Fig. 4b). No microhemorrhage and cytoarchitectural changes between sonicated tumor sections and nonsonicated tumor sections were observed within the timeframe above.

### Sonobiopsy did not induce evident inflammation/immune responses within a short time period

**Fig. 5:**
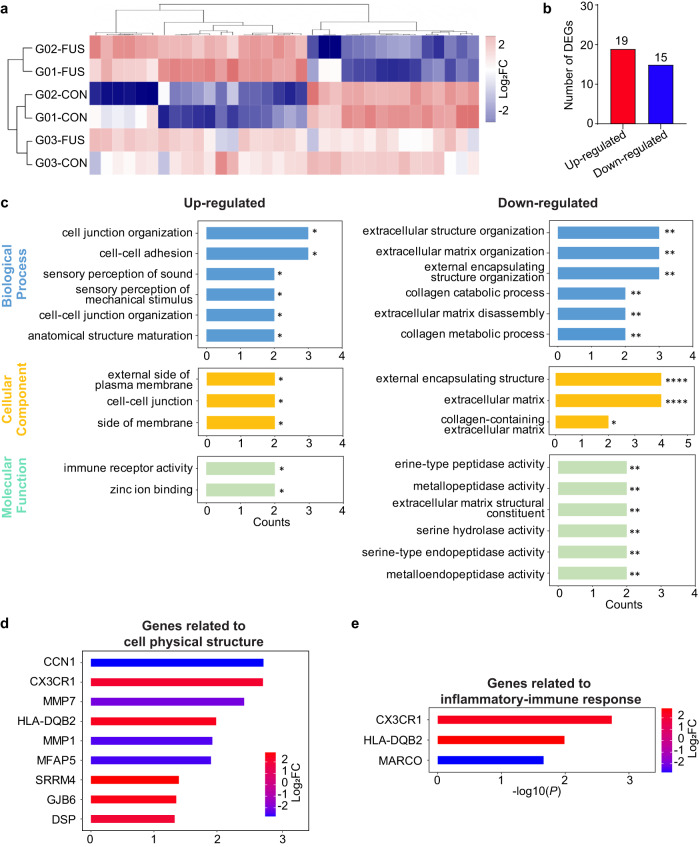
Transcriptome analysis of differentially expressed genes (DEGs) after sonobiopsy. **a** Hierarchical clustering heat map of all DEGs with log2FC (fold change) values > 2 and *p* < 0.05. Rows represent brain tissue samples acquired from the FUS-sonicated (FUS) or nonsonicated (CON) tumor regions from different patients, and columns represent individual DEGs. **b** Summary of the total number of upregulated and downregulated DEGs. **c** Upregulated (red) and downregulated (blue) DEGs with GO functional analysis for enriched biological processes, cellular components, and molecular functions. *p < 0.05, **p < 0.01, ***p < 0.001, and ****p < 0.0001. **d** Upregulated and downregulated genes involved in GO terms related to cell physical structure. **e** Upregulated and downregulated genes involved in GO terms related to inflammatory or immune-related response.

We conducted transcriptome analysis of sonicated and nonsonicated tumor tissues from G01, G02, and G03. These tissue samples were collected within 1.7 ± 0.4 h after FUS sonication. We identified differentially expressed genes (DEGs) using hierarchical clustering analysis following strict criteria of the absolute value of log2 (fold-change) >2 and *P*-value < 0.05 (Fig. 5a). Our analysis identified 34 DEGs out of 17,982 total identified genes (0.19%), among which 19 transcripts were identified as upregulated DEGs associated with sonication and 15 were identified as downregulated DEGs (Fig. 5b).

The gene ontology (GO) analysis of the upregulated and downregulated DEGs showed that the enriched GO terms were related to the physical structures of cells, including their interactions with neighboring cells and their surrounding extracellular matrix (Fig. 5c). This suggests that FUS combined with microbubbles caused mechanical perturbation to the cell-cell and cell-matrix interaction. The genes related to cell physical structure that were upregulated and downregulated were further summarized in Fig. 5d. Quantitative real-time PCR (qRT-PCR) analysis was performed to verify these genes’ upregulation and downregulation (Supplementary Figure 3).

Previous studies reported FUS with microbubbles to induce sterile inflammation in healthy mouse brains^35–37^. However, it is noteworthy that among the top 26 enriched GO terms, only one immune-related GO term was identified—“immune receptor activity GO0140375” (Fig. 5c). Out of 17,982 genes, only two upregulated differentially expressed genes, namely CX3CR1 and HLA-DQB2, were found to be associated with the immune/inflammatory-response pathway (Fig. 5e). Only one downregulated differentially gene, MARCO, was found in this pathway. The upregulation of CX3CR1 and downregulation of MARCO were further confirmed by qRT-PCR (Supplementary Figure 3). The transcriptome analysis was not performed in the last 2 patients (G04 and G05) because RNA sequencing analysis of sonicated and nonsonicated tumor tissues from the initial 3 patients already demonstrated that sonobiopsy did not induce evident immune/inflammatory-response.

## Discussion

We present the first prospective clinical study of sonobiopsy in high-grade glioma patients. Our study utilized the nimble sonobiopsy device, which was seamlessly integrated with an existing clinical neuronavigation system. The sonobiopsy procedure was performed using an established clinical workflow for neuronavigation. Our findings provide crucial initial evidence that sonobiopsy can enrich brain tumor biomarkers by targeting specific tumor locations and coordinating the blood collection time.

We demonstrated significant technological advancements that the sonobiopsy device offers to adopt this innovative technique in the clinic. The nimble design of the FUS device allowed for direct attachment of the FUS device to a neuronavigation probe used by any clinical neuronavigation system, enabling precise positioning of the FUS transducer with high accuracy. Furthermore, this unique design allowed for easy integration of sonobiopsy into the existing clinical workflow, eliminating the need for additional training of neurosurgeons to perform the sonobiopsy procedure. This will reduce the barrier to adopting this technique in the future. Of significant importance for future considerations is the fact that an operating room is not necessary. While cranial fixation and anesthesia were used for the patients described in this work, it is not essential. Standard navigation techniques that can be used without fixation and anesthesia could enable sonobiopsy to be performed outside classic operative and procedural environments (e.g., hospital rooms and clinics). The sonobiopsy device also incorporated numerical simulation of the acoustic energy delivered into the brain. The simulation provided critical guidance for selecting FUS parameters and allowed visualization of the ultrasound beam shape and location inside the brain.

We demonstrated that sonobiopsy could be integrated with advanced blood-based biomarker analysis techniques for the noninvasive and spatially targeted molecular diagnosis of brain tumors without causing tissue damage. Sonobiopsy enriched the plasma level of mononucleosome cfDNA fragment (120–280 bp), patient-specific tumor variant ctDNA, and TERT mutation ctDNA. A significant increase in cfDNA level was detected in 4 out of 5 patients. ctDNA-based sequencing assays can be divided into two classes: tumor-naive assays and tumor-informed assays. Tumor-naive assays use broad panel-based sequencing assays for genotyping or tumor early detection with a detection limit of about 0.2%^38^; Tumor-informed assays are designed in reference to mutations known from the tumor and can reach a limit of detection as low as 0.01% variant allele frequency^38^. Examples of tumor-informed assays include CAPP-Seq^39^, PhasED-seq^40^, and personalized tumor-specific sequencing^40^. In this study, we used the Invitae Personalized Cancer Monitoring assay, which was developed to detect residual molecular diseases by sensitively detecting ctDNA in the plasma samples. Our results show that sonobiopsy significantly increased the detection of patient-specific tumor variant ctDNA in 3 out of 5 patients. The other two patients had low baseline ctDNA levels. Such low levels were close to the detection limit of the Invitae assay, which may contribute to limited sensitivity in detecting mutation level changes post-FUS. ddPCR is a targeted approach for detecting specific known mutations with high sensitivity and tissue concordance^41–43^. ddPCR was used in our study to detect ctDNA with prior knowledge of the mutations expressed by the GBM tumors. The GBM tumors are known to have TERT mutation but no IDH1 mutation. The ddPCR results demonstrate that sonobiopsy dramatically enriched the level of TERT mutation in 2 (G02 and G03) out of 4 patients who had the TERT mutantion without affecting the amount of IDH1 mutation, implying that sonobiopsy can improve the sensitivity in mutation detection without affecting its specificity. The other two patients who had the TERT mutation (G01 and G04) had lower baseline TERT mutation levels than those of the G02 and G03. Patients with low baseline levels may have fewer mutants in the tumor that can be released by sonobiopsy, leading to lower efficiency of sonobiopsy. This first-in-human prospective clinical study demonstrated the great promise of sonobiopsy in enriching patient-specific ctDNA in the plasma. Future study is needed to optimize the efficacy of sonobiopsy. The previous retrospective study by Meng et al. found that increasing the sonication volume could increase the biomarker release efficiency^30^. We selected to target a single brain location in this pilot study. Future studies could evaluate the impact of FUS sonication parameters, including sonication volume, on the efficiency of sonobiopsy to determine the optimal operation parameters.

The prospective trial design implemented in this study allowed for collecting brain tumor tissue samples from each patient’s sonicated and nonsonicated tumor regions. This approach provided an unprecedented opportunity to evaluate the bioeffects of FUS sonication on the tumor in brain tumor patients. We conducted the transcriptome analysis of FUS effects on patient brain tumors obtained at 1.7 ± 0.4 h post-FUS sonication. The differentially expressed genes were only 0.19% of total identified genes after sonication. Most upregulated and downregulated genes were related to the physical structures of cells, such as cell interactions with neighboring cells and the extracellular matrix. This finding suggests that FUS combined with microbubbles caused mechanical perturbation to cell-cell and cell-matrix interactions. Notably, the downregulation of MMP1 and MMP7 genes was previously reported to be associated with BBB integrity^44^, suggesting that the sonobiopsy procedure induced BBB opening at the targeted tumor region. FUS-induced BBB opening has been shown to induce an inflammatory response in mice^35–37^, but no immunological response was observed at 7 days after FUS treatment in GBM patients in a previous reported clinical study^20^, Our study showed that only three differentially expressed genes, CX3CR1, HLA-DQB2, and MARCO, were related to the immune response in high-grade glioma tumors obtained at 1.7 ± 0.4 h post-FUS sonication. This lack of activation of the immune response was consistent with the previous reported clinical study.

The results presented in this pilot clinical trial provide essential insights into the potential for sonobiopsy in noninvasive molecular characterization of brain tumors. Sonobiopsy has the potential to achieve several critical benefits after integration into clinical practice as a complement to neuroimaging and tissue biopsy, including the identification of genetic features before surgical intervention, enabling alterations in surgical strategy. It could also enable the rapid determination of the molecular identity of suspicious lesions observed on neuroimaging scans, particularly in patients who are poor surgical candidates. Furthermore, the ability to repeatedly sample and monitor tumor recurrence and treatment response could provide valuable information to clinicians. In challenging situations where assessment based on neuroimaging alone remains difficult, such as distinguishing treatment-induced pseudoprogression from true relapse, sonobiopsy could provide complementary information. Moreover, it has the potential to support investigations into tumor-specific molecular mechanisms driving disease and accelerate the development of new treatment strategies.

While this study presents milestone achievements in developing sonobiopsy for the molecular diagnosis of brain tumors, several limitations exist. First, although the data were extremely promising to show the feasibility and safety of sonobiopsy, this pilot study was performed with five high-grade glioma patients. Further studies with larger sample sizes are needed to confirm these initial findings and establish the clinical utility of sonobiopsy. Second, this pilot study revealed the kinetics of biomarker changes post-FUS within the 5–30 min. Future studies are needed to reveal the complete kinetics of biomarker release and determine the optimal blood collection time. Third, there is always a spatial shift in the brain during the surgical dissection of the sonication brain tumor tissue. This potential shift could have introduced an error in the localization of the FUS-sonicated tumor region. To reduce the potential impact of this error, the targeted tumor region was selected to be located at the relative superficial tumor location so that this region was encountered early in the surgery and before substantial brain shift.

In conclusion, this study marks a crucial initial milestone in demonstrating the feasibility and safety of sonobiopsy in patients with high-grade glioma. This innovative technique allows for noninvasive, spatially targeted, and temporally controlled detection of brain tumor-specific biomarkers in the blood. The promising feasibility and safety data obtained from this pilot study pave the way for further advancements in translating sonobiopsy into impactful diagnostics for brain tumors and other neurological disorders.

## Methods

### Study design

This prospective, single-arm, single-center, first-in-human study was designed to evaluate the feasibility and safety of sonobiopsy in patients with brain tumors. This study was approved by the Research Ethics Board at Washington University in St. Louis, School of Medicine, and was registered with ClinicalTrials.gov (NCT0528173). All subjects provided written informed consent before enrollment. This trial complied with the International Conference on Harmonization guideline for Good Clinical Practice Tri-Council Policy Statement on ethical conduct for human research (TCPS-2).

### Sonobiopsy device

A FUS transducer consisting of 15 concentric individual ring transducers with a center frequency of 650 kHz (Imasonics, Voray-sur-l’Ognon, France) was used. The aperture of the transducer was 65 mm, and the focal distance was 65 mm (f-number = 1). As reported in our previous study^45^, the axial and lateral full width at half maximums (FWHM) of the FUS transducer were 20 mm and 3.0 mm, respectively. The FUS transducer was integrated with a passive acoustic detector at its center. The FUS transducer was driven by a commercial FUS system (Image Guided Therapy, Pessac, France). The transducer was coupled to the passive blunt probe (Stealth S8, Medtronic) through a customized adapter. The adapter was attached to the back of the FUS transducer and connected to a cylinder aligned with the FUS transducer’s central axis. The diameter of the cylinder matched that of the neuronavigation probe. This adapter design leveraged the light weight of the FUS transducer and mechanically co-aligned the neuronavigation tracker to the central axis of the transducer. The location of the FUS focus was calibrated to be 80 mm from the tip of the passive blunt probe along the probe’s trajectory. An 80 mm offset was added in the neuronavigation software so that the tip of the “virtual probe” indicated the location of the FUS focus.

### Sonobiopsy clinical workflow

The overall workflow of this clinical study is summarized in Fig. 1d. It consists of four main steps: treatment planning, patient preparation, FUS sonication, and blood and tissue collection.

Step 1: Treatment planning. CT and MRI images of the patient’s head were acquired a few days before the procedure. FUS sonication trajectory was planned using the Medtronic S8 planning station (Medtronic Plc, Dublin, Ireland). The trajectory was selected using the following criteria: close to 90° incident angle (best effort), focus depth below skin <35 mm (limited by the focal length of our FUS transducer), and avoiding ultrasound beam passing through the ear lobe and eye. A full-wave acoustic simulation using the k-Wave toolbox was performed to estimate the ultrasound pressure field distribution inside the brain and calculate the skull attenuation using methods reported in our previous publication^45^.

Step 2: Patient preparation. On the day of the procedure, the Mayfield skull clamp (Integra LifeSciences, Princeton, NJ) was fixed to the patient’s head under local and general anesthesia. The skull clamp was connected to the surgical table through a Mayfield bed attachment. The stealth arc and Veltek arm were connected, and the patient’s head was registered to the pre-acquired MRI/CT images. The planned FUS trajectory was then entered into the neuronavigation system. A small patch of hair above the tumor region was shaved, and the exposed skin was thoroughly cleaned with alcohol pads. Deionized water was filled into the transducer water bladder, continuously degassed with a degassing system for more than 15 min. Degassed ultrasound gel was applied liberally to the exposed skin area. The FUS transducer was then placed on the patient’s head under the guidance of the neuronavigation system.

Step 3: FUS sonication. The passive cavitation detector was used to check the quality of the acoustic coupling between the FUS transducer and the skin. If broadband emissions were present in the detect signals when the FUS was turned on without microbubble injection, the most likely cause was due to air bubbles trapped in the coupling media. In this case, we would remove the FUS transducer, clean it, and re-apply the ultrasound gel. The input electrical power was determined based on hydrophone calibration of the FUS transducer focal pressure over different input electrical powers derated by the skull attenuation estimated in Step 1 based on k-wave simulation. To ensure the safety of this study, the estimated in situ acoustic pressure was selected to ensure the mechanical index (MI) was below 0.8, consistent with other FUS-BBBD drug delivery clinical studies^20,46^. Acoustic parameters besides input power were selected to be the same as our previous preclinical work^29^. The FUS parameters were: center frequency = 650 kHz (f_0_); pulse repetition frequency = 1 Hz; pulse duration = 10 ms; treatment duration = 3 min. Fifteen seconds after FUS sonication began, microbubbles (Definity, Lantheus Medical Imaging, North Billerica, MA) were administered intravenously by the standing anesthesiologist at a dose of 10 µL/kg body weight diluted with saline and followed with a saline flush. The injection rate was controlled with the best effort to be 10 s/mL, recommended by the manufacturer. In reference to our previous publication^47^, a custom MATLAB script was written to process the acquired cavitation data to evaluate the stable cavitation and inertial cavitation levels. Briefly, the stable and inertial cavitation levels were calculated as the root-mean-squared amplitudes of subharmonic (f_0_/2 ± 0.15 MHz) and broadband (0.3–2 MHz after removing f_0_/2 ± 0.15 MHz and *n*f_0_ ± 0.15 MHz where *n* = 1, 2, 3) signals, respectively.

Step 4: Blood and tissue collection. Blood samples (20 mL each) were collected 5 mins before and within 30 mins after FUS sonication. Blood samples were stored in BD Vacutainer® EDTA (BD Biosciences, San Jose, CA) tubes or Cell-Free DNA BCT (Streck Laboratories, La Vista, NE) tubes. Within 4 h of collection, whole blood samples were centrifuged at 1200 × *g* for 10 mins at 4 °C. Isolated plasma was centrifuged a second time at 1800 × *g* for 5 mins at 4 °C to further remove cell debris. Plasma aliquots were immediately put on dry ice for snap freezing and stored at −80 °C for later downstream analysis. The plasma-depleted whole blood cells were stored as well for tissue sequencing analysis. After blood collection, craniotomy was performed and the tumor was resected under the guidance of the neuronavigation system. Sonicated and nonsonicated part of tumor tissue was collected from the resected tumor. Skin tissue on the trajectory of FUS sonication was also collected during surgery. The collected tissues were fixed in formalin for paraffin embedding or put in a fresh medium for snap freezing.

### Cell-free DNA extraction and quantification

Maxwell® HT ccfDNA Kit (Promega, Madison, WI) and KingFisher Flex (Thermo Fisher Scientific, Waltham, MA) were used to extract cfDNA from patient plasma per the manufacturer’s protocol (Invitae, San Francisco, CA). cfDNA was eluted in 110 µL of each corresponding buffer and was quantified using Qubit Fluorometric Quantitation (Thermo Fisher Scientific, Waltham, MA). The 2100 Bioanalyzer (Agilent Technologies, Santa Clara, CA) was used to assess the size distribution and concentration of cfDNA extracted from plasma samples. The cfDNA in the mononucleosome size range (120–180 bp) was determined with the software as the area under the peaks^48^.

### Personalized tumor-informed ctDNA assay

Invitae Personalized Cancer Monitoring (PCM) was adopted to detect the patient-specific tumor variants in patients’ plasmas in the following three steps (Invitae, San Francisco, CA). First, the data from whole exome sequencing (WES) on tumor and normal (peripheral blood, PB) samples were processed using Invitae’s WES Pipeline. The variants identified from the tumor and normal samples were then compared to identify patient-specific tumor variants. Variant calls were used as input for the minimal residual disease (MRD) Panel Designer pipeline. Second, patient-specific panels (PSPs) were designed to target up to 50 patient-specific single tumor variants. The Panel Designer identified high-confidence patient-specific tumor variants which could be targeted using an Anchored Multiplex PCR (AMP) panel. Third, cfDNAs were extracted from the patient’s plasma samples and used as input for an AMP library preparation using the personalized panel designed for the patient. Libraries were sequenced using the NovaSeq 6000 sequencing platform (Illumina, San Diego, CA) and the resulting fastq files were analyzed using the Invitae MRD calling pipeline. The MRD analysis pipeline aligns MRD library sequences to the genome, calculates the error rates for the targeted variants and measures the allele fraction for the targeted variants. The observed allele fractions are compared to the background error rate to determine the MRD call. To calculate the concentrations of patient-specific tumor variants in each plasma, the value of alternative observations (AOs) from the Inviate MRD was normalized to the input volume of plasma (tumor variant ctDNA copies/ml plasma).

### ddPCR assays

Custom sequence-specific primers and fluorescent probes were designed and synthesized for patient-specific variant detection (Sigma Aldrich, St. Louis, MO). The forward and reverse primer and probe sequences are listed in Supplementary Table 4. ddPCR reactions were prepared with 2 × ddPCR Supermix for probes (no dUTP) (Bio-Rad, Hercules, CA), 2 µL of target cfDNA product, 0.1 µM forward and reverse primers, and 0.1 µM probes. Alternatively, 100 µM 7-deaza-dGTP (New England Biolabs, Ipswich, MA) was added to improve PCR amplification for GC-rich regions for TERT promoter. The QX200 manual droplet generator (Bio-Rad, Hercules, CA) was used to generate droplets. The PCR step was performed on a C1000 Touch Thermal Cycler (Bio-Rad, Hercules, CA) by use of the following program: 1 cycle at 95 °C for 10 min, 48 cycles at 95 °C for 30 s and 60 °C for 1 min, 1 cycle at 98 °C for 10 mins, and 1 cycle at 4 °C infinite, all at a ramp rate of 2 °C/s. All plasma samples were analyzed in technical duplicate or triplicate based on sample availability. Data were acquired on the QX200 droplet reader (Bio-Rad, Hercules, CA) and analyzed using QuantaSoft Analysis Pro (Bio-Rad, Hercules, CA). All results were manually reviewed for false positives and background noise droplets based on negative and positive control samples. Tumor mutation ctDNA concentrations (copies/ml plasma) were calculated by multiplying the normalized tumor mutation copies (the tumor mutation copies obtained from QuantaSoft were divided by the input cfDNA volume) by the total cfDNA volume, divided by the input plasma volume used during cfDNA extraction. The minimal detection is 1 copy/ddPCR reaction using 2.2 µL cfDNA from total 110 µL cfDNA of 5.0 ml plasma. Therefore, 10 copies/ml plasma of tumor variant ctDNA is the detection limit for our ddPCR.

### Bulk RNA sequencing and analysis

Bulk RNA sequencing and analysis were conducted at Genome Technology Access Center at the McDonnell Genome Institute at Washington University in St. Louis. According to the manufacturer’s instructions, snap-frozen sonicated and nonsonicated tumor tissues were homogenized and isolated using the RNeasy MiniPlus Kit (Qiagen, Hilden, Germany). Total RNA integrity was determined using Agilent Bioanalyzer (Agilent Technologies, Santa Clara, CA). Ribosomal RNA was removed by a hybridization method using Ribo-ZERO kits (Illumina-EpiCentre, San Diego, CA). mRNA was reverse transcribed to yield cDNA using SuperScript III RT enzyme (Thermo Fisher Scientific, Waltham, MA) and random hexamers. A second strand reaction was performed to yield double-stranded cDNA. cDNA was blunt-ended, had an A base added to the 3′ ends, and then had Illumina sequencing adapters ligated to the ends. Ligated fragments were then amplified for 12–15 cycles using primers incorporating unique dual index tags. Fragments were sequenced on an Illumina NovaSeq-6000 (Illumina, San Diego, CA) using paired-end reads extending 150 bases. Basecalls and demultiplexing were performed with Illumina’s bcl2fastq2 software. RNA-seq reads were then aligned and quantitated to the Ensembl release 101 primary assembly with an Illumina DRAGEN Bio-IT on-premise server running version 3.9.3-8 software.

All gene counts were then imported into the R/Bioconductor package EdgeR and TMM normalization size factors were calculated to adjust for samples for differences in library size. Ribosomal genes and genes not expressed in the smallest group size minus one sample greater than one count per million were excluded from further analysis. The TMM size factors and the matrix of counts were then imported into the R/Bioconductor package Limma. Weighted likelihoods based on the observed mean-variance relationship of every gene and sample were then calculated for all samples, and the count matrix was transformed to moderated log 2 counts-per-million with Limma’s voomWithQualityWeights. The performance of all genes was assessed with plots of the residual standard deviation of every gene to their average log count with a robustly fitted trend line of the residuals. Differential expression analysis was then performed to analyze for differences between conditions, and the results were filtered for only those genes with Benjamini-Hochberg false-discovery rate adjusted *p* values less than or equal to 0.05.

To identify differentially expressed genes (DEGs), we applied strict criteria: log2 (fold-change) >2 and *p* < 0.05 for upregulated genes and log2 (fold-change) < −2 and *p* < 0.05 for downregulated genes. Gene ontology (GO) enrichment analysis was performed using the g: Profiler tool (https://biit.cs.ut.ee/gprofiler/) on the DEG list classified into upregulated and downregulated lists. The results were visualized as bar plots or dot plots, which were generated in R using ggplot. Raw data were evaluated for statistical significance with a threshold of *p* < 0.05, using an independent *t*-test to compare fold changes.

### Histological analysis

Brain tumor tissues from sonicated and nonsonicated regions were resected and fixed in formalin for paraffin embedding. The brain tumor tissue samples were sectioned into 10 μm slices for hematoxylin and eosin (H&E) staining to examine red blood cell extravasation and tissue damage. Digital images of tissue sections were obtained using an all-in-one microscope (Keyence Corporation of America, Itasca, IL). An experienced clinical neuropathologist assessed histological images.

### Quantitative real-time PCR (qRT-PCR)

Total RNA was isolated from fresh-frozen sonicated tumor and nonsonicated tumor using the RNeasy Plus Mini Kit (Qiagen) and cDNA was synthesized using High-Capacity cDNA Reverse Transcription Kit (ThermoFisher Scientific, Waltham, MA). Quantitative real-time PCR (qRT-PCR) was performed with SYBR Select Master Mix (Applied Biosystems, Waltham, MA) on a 7900HT Real-Time PCR System (Applied Biosystems, Waltham, MA). HMBS was used as the endogenous amplification control. Primer sequences are listed in Supplementary Table 5. Data processing was performed using SDS 2.4 software (Applied Biosystems, Waltham, MA), and the relative quantitation of the expression level of each mRNA was performed using the comparative CT method (2-ΔΔCT).

### Statistical analysis

Statistics analysis was performed in Graphpad (Prism) (Graphpad, Boston, MA). Ordinary one-way ANOVA, uncorrected Fishers’ LSD test was used to compare the level of cfDNA in post-FUS plasma with that in pre-FUS plasma. Repeated measures (RM) one-way ANOVA, uncorrected Fishers’ LSD test was conducted to compare the concentrations of tumor variant ctDNA in post-FUS plasmas with that in pre-FUS plasma. An independent *t*-test was used to compare fold changes in gene expression levels in post-FUS plasma with pre-FUS plasma. All reported *p* values are two-tailed unless otherwise specified.

### Reporting summary

Further information on research design is available in the Nature Research Reporting Summary linked to this article.

### Supplementary information

Supplementary Information

Reporting Summary

## Data Availability

The bulk RNA sequencing and whole exome sequencing data of patient brain tumor in this study have been deposited in the NCBI SRA (PRJNA1009135). All relevant data are available from the corresponding authors upon reasonable request.

## Code availability

All relevant codes are available from the corresponding authors upon reasonable request.
