# Supplementary material for: First-in-human prospective trial of sonobiopsy in high-grade glioma patients using neuronavigation-guided focused ultrasound
Source: NPJ Precis Oncol. 2023 Sep 16;7:92. doi: 10.1038/s41698-023-00448-y (PMC10505140; doi:10.1038/s41698-023-00448-y)
Supplement: Supplementary file 1 — Supplementary Information [file 41698_2023_448_MOESM1_ESM.pdf]

Supplementary Table 1. Patient demographics

Patient	Sex	Age range	Diagnosis	IDH1	TERT
G01	M	73	Glioblastoma	Wild type	TERT C228T
G02	M	58	Glioblastoma	Wild type	TERT C250T
G03	F	66	Glioblastoma	Wild type	TERT C228T
G04	M	66	Glioblastoma	Wild type	TERT C250T
G05	M	36	Diffuse high-grade glioma	Wild type	Wild type

Supplementary Table 2. Inclusion and exclusion criteria

Inclusion Criteria:

- Must be newly diagnosed with a lesion in the brain with imaging characteristics consistent with high-grade glioma. Scan must have occurred no more than 28 days prior to enrollment.
- Lesion must be > 3 cm in maximal dimension on MRI.
- Lesion must be in the supratentorial space within 5 cm of the cortical surface.
- Lesion must be gadolinium enhancing.
- Low grade tumors and metastatic tumors
- Recurrent brain tumors and/or radiation necrosis
- Must be planning to undergo surgical resection of the tumor.
- Must be at least 18 years old.

Exclusion Criteria:

- Contraindication to MRI.
- Previous cranial surgery.
- Previous history of cancer and/or cancer treatments.
- Coagulopathy within 14 days of enrollment defined as PT/PTT outside of normal parameters and platelets < 100,000/mcL.
- Physical skull defect of any kind.
- Ferrous material in the scalp or skull.
- Scalp or skin disease that limits contact with the ultrasound probe.
- Enrolled in another clinical trial where intervention is administered prior to surgery.
- Known hypersensitivity to polyethylene glycol.
- Known unstable cardiopulmonary condition (e.g. acute myocardial infarction, acute coronary artery syndromes, worsening or unstable congestive heart failure, serious ventricular arrhythmias).

Supplementary Table 3. Summary of sonobiopsy parameters

Patient	G01	G02	G03	G04	G05
Simulated in situ pressure (MPa)	0.47	0.31	0.64	0.53	0.40
Estimated mechanical index (MI)	0.58	0.38	0.80	0.66	0.50
Procedure duration from the time when the patient was ready to the end of FUS sonication (min)	24	14	30	23	20
Microbubble dose (µL/Kg)	10	10	10	10	10
Stable cavitation dose (a.u.)	4.08	1.81	7.77	20.6	4.11
Inertial cavitation dose (a.u.)	0.47	0.25	80.7	150.9	0.68

Supplementary Table 4. Primers and probes used for ddPCR assays

Variant	TERT C228T	TERT C250T	IDH1 R132H
Mutant probe	(6-FAM) 5’-AGCCCCTTCCGGGCCCTCCCA-3’ (BHQ1)	(6-FAM) 5’-CGTCCCGACCCCTTCCGGGT-3’ (BHQ1)	(6-FAM) 5’CATCATAGGTCATCATGCTTATGGGG -3’ (BHQ1)
Wild type probe	(HEX) 5’-AGCCCCCTCCGGGCCCTCCCA-3’ (BHQ1)	(HEX) 5’-CGTCCCGACCCCTCCCGGGT-3’ (BHQ1)	(HEX) 5’CATCATAGGTCGTCATGCTTATGGGG-3’ (BHQ1)
Forward primer	5'-TCCAGCTCCGCCTCCTCC-3'	5'-TTCCAGCTCCGCCTCCTCC-3'	5'-GGCTTGTGAGTGGATGGG-3'
Reverse primer	5'-AGCGCTGCCTGAAACTCGC-3'	5'-AGCGCTGCCTGAAACTCGC-3'	5'-ACACATACAAGTTGGAAATTTCTGG-3'

Supplementary Table 5. Primers used for qRT-qPCR assays

Gene	Forward primers	Reverse primer
DSP	5’-GCTGAACATACCTATCAAGAGGACC-3’	5’-TCTTCAGCATCTCACTTAAGAACCTG-3’
GJB6	5’- CAGCTGATCTTCGTCTCCACC-3’	5’- GATGCTGCTGGTGTACGTCC-3’
MMP1	5’- ACATTCACCAAGGTCTCTGAGG-3’	5’- CGAGTTCATGAGCTGCAACACG-3’
MMP7	5’- ACATTCACCAAGGTCTCTGAGG -3’	5’- CGAGTTCATGAGCTGCAACACG-3’
CCN1	5’- GACAGCCAGTGTACAGCAGC-3’	5’- TCACAGTCCTGGTCAGCTGG-3’
MFAP5	5’- GCACCTTCCACAGATGACTTGG-3’	5’- AGACAAGACGAGAGCAGATCTCC-3’
CX3CR1	5’-TGCTACTTCAGAATCATCCAGACG-3’	5’- TCCTTCCTCATGTCACAACTGG-3’
MARCO	5’- CATGAGCACTTGCTGCAGC-3’	5’- CCTTCTCAGCAGGTGGTCC-3’
HMBS	5’- ATCATCCTGGCAACAGCTGG -3’	5’- CTTGGCTCGCACTTCCACG-3’

Supplementary Figures and Legends

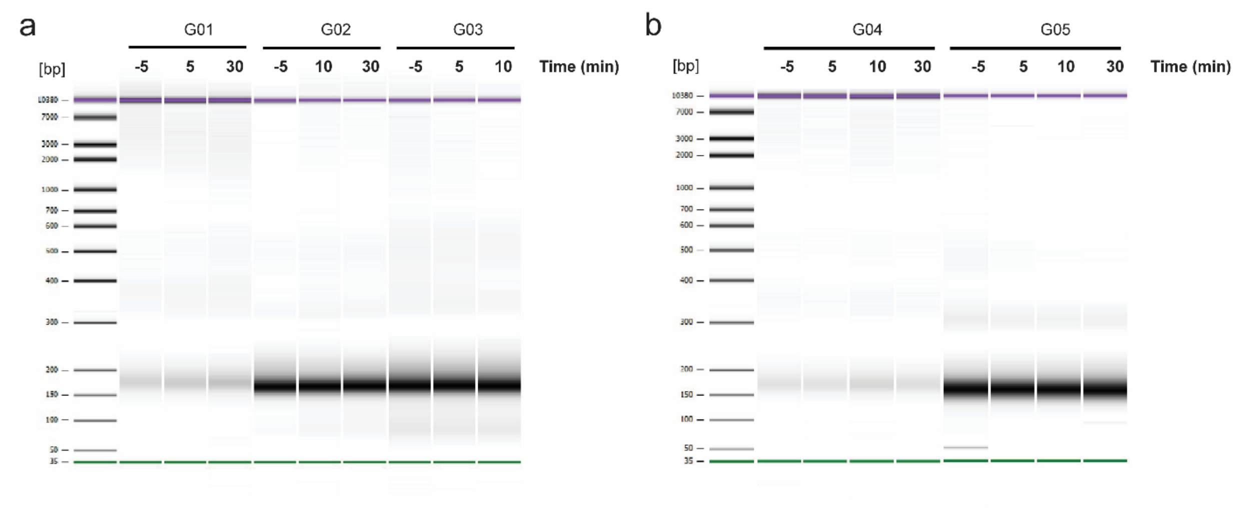

Supplementary Figure 1. Plasma cfDNA quantity analysis.

Bioanalyzer DNA gel analysis of plasma cfDNA from patient G01, G02, G03 **a** and G04, G05 **b** used for personalized tumor-informed ctDNA assays and ddPCR assays. The peak size around 160bp representing mononucleosomal cfDNA clearly presented in each cfDNA sample.

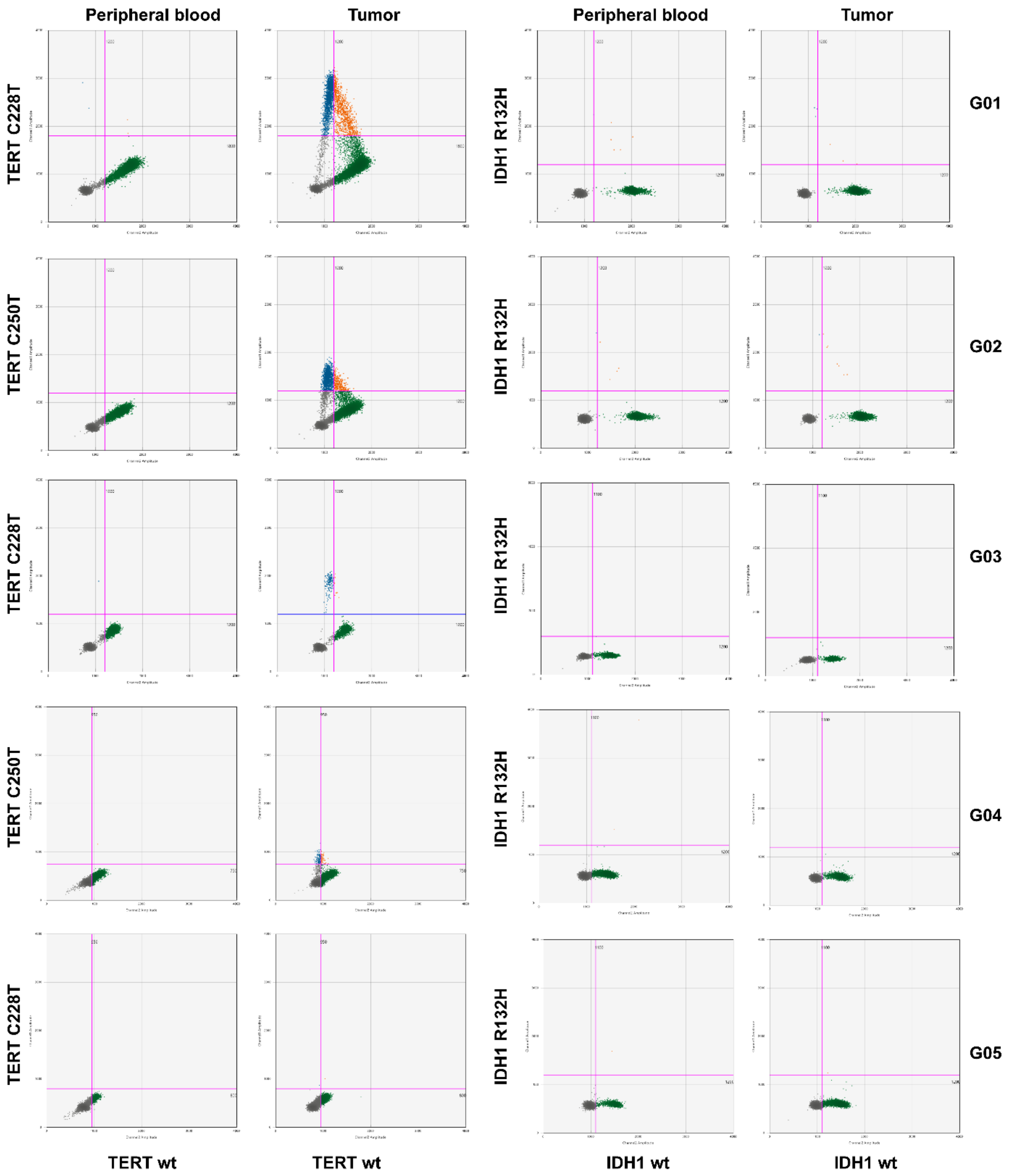

Supplementary Figure 2. Evaluation of TERT promoter and IDH1 mutation for five patients with ddPCR assays.

Four patients are positive for TERT promoter mutation and all five patients are negative for IDH1 R132H mutaion.

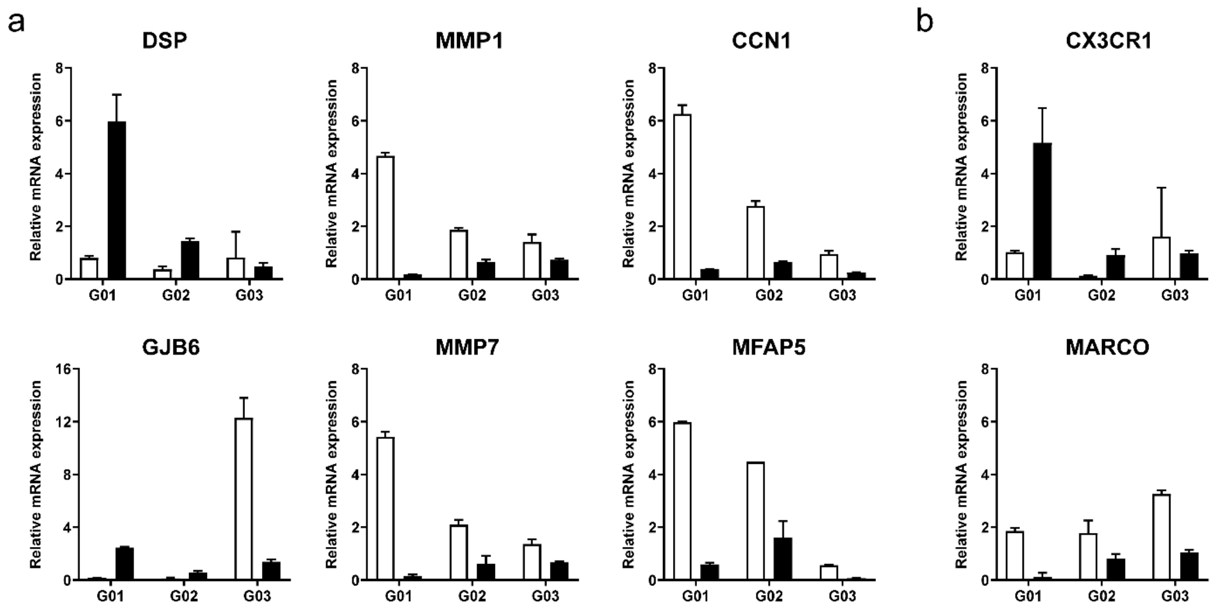

Supplementary Figure 3. Analysis of gene expression changes by FUS sonication using qRT-PCR.

a The relative mRNA expression for genes associated with cellular interactions after FUS sonication. **b** The relative mRNA expression for genes associated with inflammatory response after FUS sonication. Open bar represents nonsonicated tumor tissue (CON) and solid bar represents sonicated tumor tissue (FUS). The error bars show the standard deviation for the replicates in qRT-PCR.
