# Supplementary material for: First-in-human prospective trial of sonobiopsy in high-grade glioma patients using neuronavigation-guided focused ultrasound
Source: NPJ Precis Oncol. 2023 Sep 16;7:92. doi: 10.1038/s41698-023-00448-y (PMC10505140; doi:10.1038/s41698-023-00448-y)
Supplement: Supplementary file 2 — Reporting Summary [file 41698_2023_448_MOESM2_ESM.pdf]

nature portfolio

Corresponding author(s): Hong Chen; Eric C. Leuthardt

Last updated by author(s): 08/28/2023

Reporting Summary

Nature Portfolio wishes to improve the reproducibility of the work that we publish. This form provides structure for consistency and transparency in reporting. For further information on Nature Portfolio policies, see our Editorial Policies and the Editorial Policy Checklist.

Statistics

For all statistical analyses, confirm that the following items are present in the figure legend, table legend, main text, or Methods section.

n/a	Confirmed	
☐	☒	The exact sample size (n) for each experimental group/condition, given as a discrete number and unit of measurement
☐	☒	A statement on whether measurements were taken from distinct samples or whether the same sample was measured repeatedly
☐	☒	The statistical test(s) used AND whether they are one- or two-sided<br>*Only common tests should be described solely by name; describe more complex techniques in the Methods section.*
☒	☐	A description of all covariates tested
☐	☒	A description of any assumptions or corrections, such as tests of normality and adjustment for multiple comparisons
☐	☒	A full description of the statistical parameters including central tendency (e.g. means) or other basic estimates (e.g. regression coefficient) AND variation (e.g. standard deviation) or associated estimates of uncertainty (e.g. confidence intervals)
☒	☐	For null hypothesis testing, the test statistic (e.g. F, t, r) with confidence intervals, effect sizes, degrees of freedom and P value noted<br>*Give P values as exact values whenever suitable.*
☒	☐	For Bayesian analysis, information on the choice of priors and Markov chain Monte Carlo settings
☒	☐	For hierarchical and complex designs, identification of the appropriate level for tests and full reporting of outcomes
☒	☐	Estimates of effect sizes (e.g. Cohen's d, Pearson's r), indicating how they were calculated

Our web collection on statistics for biologists contains articles on many of the points above.

Software and code

Policy information about availability of computer code

Data collection	The k-Wave toolbox was performed to estimate the ultrasound pressure field distribution inside the brain and calculate the skull attenuation; 2100 Bioanalyzer was used to collect the data of plasma cfDNA; Invitae PCM/MRD was used to collect the data of personalize plasma tumor variant ctDNA; QuantaSoft was used to collect ddPCR data of plasma ctDNA; Illumina DRAGEN Bio-IT was used to collect the bulk-RNA-Seq data of tumor tissues; Keyence BZ-X800 was used to take histology images of tumor tissues
Data analysis	MATLAB (R2021a) was used to analyze the stable and inertial cavitation dose of microbubbles, 2100 Expert was used analyze the 120-280bp cfDNA; Invitae PCM/MRD was used to analyze the personalize plasma tumor variant counts; QuantaSoft was used to analyze the plasma tumor variant concentrations, g: Profiler was used for GO enrichment analysis of RNA-Seq; Graphpad (Prism) and Microsoft Excel were used for statistic analysis

For manuscripts utilizing custom algorithms or software that are central to the research but not yet described in published literature, software must be made available to editors and reviewers. We strongly encourage code deposition in a community repository (e.g. GitHub). See the Nature Portfolio guidelines for submitting code & software for further information.

Data

Policy information about availability of data

All manuscripts must include a data availability statement. This statement should provide the following information, where applicable:

- Accession codes, unique identifiers, or web links for publicly available datasets
- A description of any restrictions on data availability
- For clinical datasets or third party data, please ensure that the statement adheres to our policy

Brain tumor transcriptome data in this study has been deposited in the NCBI SRA (PRJNA1009135). All relevant data are available from the corresponding authors upon reasonable request.

Research involving human participants, their data, or biological material

Policy information about studies with human participants or human data. See also policy information about sex, gender (identity/presentation), and sexual orientation and race, ethnicity and racism.

Reporting on sex and gender	Sex information was collected and reported
Reporting on race, ethnicity, or other socially relevant groupings	n/a
Population characteristics	Described in supplementary information
Recruitment	Written informed consent was obtained from all participants before study enrollment. Patients with a lesion in the brain with imaging characteristics consistent with a high-grade glioma were screened for the clinical trial.
Ethics oversight	Research Ethics Board at Washington University in St. Louis, School of Medicine

Note that full information on the approval of the study protocol must also be provided in the manuscript.

Field-specific reporting

Please select the one below that is the best fit for your research. If you are not sure, read the appropriate sections before making your selection.

☒ Life sciences ☐ Behavioural & social sciences ☐ Ecological, evolutionary & environmental sciences

For a reference copy of the document with all sections, see nature.com/documents/nr-reporting-summary-flat.pdf

Life sciences study design

All studies must disclose on these points even when the disclosure is negative.

Sample size	up to date 5 patients
Data exclusions	n/a
Replication	n/a
Randomization	All enrolled patients went through the sonobiopsy procedure.
Blinding	Invitae PCM/MRD personalized ctDNA assay and histology evaluation were blindly conducted.

Reporting for specific materials, systems and methods

We require information from authors about some types of materials, experimental systems and methods used in many studies. Here, indicate whether each material, system or method listed is relevant to your study. If you are not sure if a list item applies to your research, read the appropriate section before selecting a response.

Materials & experimental systems

n/a	Involved in the study
☒	☐ Antibodies
☒	☐ Eukaryotic cell lines
☒	☐ Palaeontology and archaeology
☒	☐ Animals and other organisms
☐	☒ Clinical data
☒	☐ Dual use research of concern
☒	☐ Plants

Methods

n/a	Involved in the study
☒	☐ ChIP-seq
☒	☐ Flow cytometry
☐	☒ MRI-based neuroimaging

Clinical data

Policy information about clinical studies

All manuscripts should comply with the ICMJE guidelines for publication of clinical research and a completed CONSORT checklist must be included with all submissions.

Clinical trial registration	Clinicaltrials.gov (http://www.clinicaltrials.gov)
Study protocol	NCT05281731
Data collection	Blood and tumor tissue were collected at Washington University School of Medicine from 4/18/2022-3/07/2023
Outcomes	Feasibility of sonobiopsy as measured the change of plasma ctDNA level and safety of sonobiopsy as examined with histology staining

Magnetic resonance imaging

Experimental design

Design type	Standard anatomical imaging
Design specifications	n/a
Behavioral performance measures	n/a

Acquisition

Imaging type(s)	T1 weighted
Field strength	3T
Sequence & imaging parameters	TR: 1700, TE: 2.39;
Area of acquisition	brain

Diffusion MRI ☐ Used ☒ Not used

Preprocessing

Preprocessing software	n/a
Normalization	n/a
Normalization template	n/a
Noise and artifact removal	n/a
Volume censoring	n/a

Statistical modeling & inference

Model type and settings	n/a
Effect(s) tested	n/a

Specify type of analysis: ☐ Whole brain ☐ ROI-based ☐ Both

Statistic type for inference (See Eklund et al. 2016): n/a

Correction: n/a

Models & analysis

n/a	Involved in the study
☒	☐ Functional and/or effective connectivity
☒	☐ Graph analysis
☒	☐ Multivariate modeling or predictive analysis
